# Regional Neural Activity Abnormalities and Whole-Brain Functional Connectivity Reorganization in Bulimia Nervosa: Evidence From Resting-State fMRI

**DOI:** 10.3389/fnins.2022.858717

**Published:** 2022-04-26

**Authors:** Jia-ni Wang, Li-rong Tang, Wei-hua Li, Xin-yu Zhang, Xiao Shao, Ping-ping Wu, Ze-mei Yang, Guo-wei Wu, Qian Chen, Zheng Wang, Peng Zhang, Zhan-jiang Li, Zhenchang Wang

**Affiliations:** ^1^Department of Radiology, Beijing Friendship Hospital, Capital Medical University, Beijing, China; ^2^Beijing Anding Hospital, Capital Medical University, Beijing, China; ^3^The National Clinical Research Center for Mental Disorders & Beijing Key Laboratory of Mental Disorders, Beijing, China; ^4^Chinese Institute for Brain Research, Beijing, China

**Keywords:** bulimia nervosa, resting-state fMRI, eating behavior, reward, cognition

## Abstract

The management of eating behavior in bulimia nervosa (BN) patients is a complex process, and BN involves activity in multiple brain regions that integrate internal and external functional information. This functional information integration occurs in brain regions involved in reward, cognition, attention, memory, emotion, smell, taste, vision and so on. Although it has been reported that resting-state brain activity in BN patients is different from that of healthy controls, the neural mechanisms remain unclear and need to be further explored. The fractional amplitude of low-frequency fluctuation (fALFF) analyses are an important data-driven method that can measure the relative contribution of low-frequency fluctuations within a specific frequency band to the whole detectable frequency range. The fALFF is well suited to reveal the strength of interregional cooperation at the single-voxel level to investigate local neuronal activity power. FC is a brain network analysis method based on the level of correlated dynamics between time series, which establishes the connection between two spatial regions of interest (ROIs) with the assistance of linear temporal correlation. Based on the psychological characteristics of patients with BN and the abnormal brain functional activities revealed by previous neuroimaging studies, in this study, we investigated alterations in regional neural activity by applying fALFF analysis and whole-brain functional connectivity (FC) in patients with BN in the resting state and to explore correlations between brain activities and eating behavior. We found that the left insula and bilateral inferior parietal lobule (IPL), as key nodes in the reorganized resting-state neural network, had altered FC with other brain regions associated with reward, emotion, cognition, memory, smell/taste, and vision-related functional processing, which may have influenced restrained eating behavior. These results could provide a further theoretical basis and potential effective targets for neuropsychological treatment in patients with BN.

## Introduction

Bulimia nervosa (BN) is a severe psychiatric disorder characterized by recurrent episodes of binge eating and purging followed by compensatory behaviors to prevent weight gain (Treasure et al., 2020), which are probably triggered by negative effects, mood lability and stress (Wierenga et al., 2014). The complex interaction of neural mechanisms and social psychology abnormalities has largely hindered the development of effective therapeutics for this disorder.

Functional magnetic resonance imaging (fMRI) provides a window to study the function and structure of the human brain, which makes it possible to clarify the interrelationships between neurological processes and eating disorders. Balodis et al. (2013) and Hege et al. (2015) using food-related Stroop color-word interference and visual go–nogo tasks emphasized the exacerbated decrease in frontal region activity, especially within the ventrolateral and dorsolateral part of the prefrontal cortex, and functional impairment was also observed in the visual cortex in obese individuals with binge eating disorder (BED). A task-based fMRI study of BN from Fischer et al. (2017) further revealed mean decreased BOLD response in response to food cues, assessed in pre- and post-stress conditions, in reward-related regions, such as the anterior cingulate cortex (ACC), amygdala, and ventromedial prefrontal cortex (vmPFC). Research has reported reduced cortical thickness in the frontal-parietal area and the left posterior cingulate cortex (PCC) in patients with BN, which supports attentional and control processes (Berner et al., 2018). A voxel-based morphometry (VBM) study evaluating brain structure reported that patients with BN showed greater gray matter volume in the orbitofrontal cortex (OFC) (Schäfer et al., 2010). Contemporary research has begun to focus on the role of structural and functional connectivity (FC) between spatially distributed brain regions. A study where BN patients received three taste stimuli found widespread alterations in white matter (WM) structure as well as effective connectivity in appetite-related and taste-reward pathways (Frank et al., 2016). Stopyra et al. (2019) found that BED patients exhibited aberrant connectivity in the dorsal ACC within the salience network (SN) and in the mPFC within the default mode network (DMN). Wang et al. (2020) observed reduced negative FC between subregions of the putamen and prefrontal and parietal areas in patients with BN.

To date, most imaging studies have used task-based fMRI to elicit neural processing associated with BN, however, the variety in task designs and results have not provided a uniform model of brain activity. There are few studies available on brain activity and FC using resting-state fMRI on the whole-brain level, and studies on FC are limited to particular brain networks or brain regions (Domakonda et al., 2019; Stopyra et al., 2019; Wang et al., 2020). To study resting-state fMRI signals, fractional amplitude of low-frequency fluctuation (fALFF) analyses are an important data-driven method that can measure the relative contribution of low-frequency fluctuations within a specific frequency band to the whole detectable frequency range (Zou et al., 2008). It is important to investigate different dimensions of resting-state functioning, such as local neuronal activity power, and fALFF analysis is well suited to reveal the strength of interregional cooperation at the single-voxel level. Furthermore, compared to the amplitude of low-frequency fluctuation (ALFF), fALFF is more sensitive to detect signals from cortical regions associated with spontaneous brain activity by selectively suppressing artifacts from non-specific signal components, such as the vicinity of large blood vessels, cisterns and ventricles (Zou et al., 2008). FC is a brain network analysis method based on the level of correlated dynamics between time series, which establishes the connection between two spatial regions of interest (ROIs) with the assistance of linear temporal correlation.

Based on the abnormal brain functional activities revealed by previous neuroimaging studies, we can definitely share the view that BN patients have changes in the functional activity and reorganization of connectivity patterns within the resting-state neural network. In this study, we applied fALFF analysis to focus on brain activation frequency differences in spontaneous fluctuations and performed a seed-based FC analysis to investigate aberrant connections between brain regions related to the disorder at the whole-brain level. We hypothesized, first, that individuals with BN would have abnormal local neural activities with resulting changes in FC between brain regions. Second, the alteration of FC patterns might involve specific brain function areas which regulate reward, emotions, cognitive control and so on, and are related to the abnormal eating behaviors of BN patients.

## Materials and Methods

### Subjects

The sample consisted of 31 BN women and 29 healthy control (HC) women. Those with BN were recruited from outpatient and inpatient services. HCs were recruited *via* local advertisements. The diagnosis of BN was made by a psychiatrist with specialized knowledge of eating disorders using the “Feeding and Eating Disorders” chapter of the Diagnostic and Statistical Manual of Mental Disorders, Fifth Edition (DSM-5) (Francesmonneris et al., 2013).

All potential participants with any history of neurologic disorder, brain injury, intellectual disability, pervasive developmental disorder, pregnancy or MRI contraindications (metal implants or claustrophobia) were excluded. In addition, blood biochemistry tests were conducted to rule out metabolic diseases such as hyperglycemia, hyperlipidemia and hyperthyroidism. Patients were required to have been free of any psychotropic medications for at least 2 months before the study. We excluded patients with current concomitant comorbid major psychiatric disorders, including anorexia nervosa, bipolar disorder, schizophrenia, major depression or anxiety disorder and alcohol or substance abuse, to avoid potentially confounding effects on the results according to the DSM-5 criteria. In addition, we conducted professional interviews with each HC using the Mini-International Neuropsychiatric Interview (MINI) (Sheehan et al., 1998) to exclude those with any psychiatric disorders.

All participants underwent MRI examinations between 8 a.m. and 11 a.m. after fasting for at least four hours. Before undergoing a magnetic resonance examination, all participants were screened using the Dutch Eating Behavior Questionnaire (DEBQ) to assess eating behavior. Previous studies have demonstrated that the DEBQ is effective for assessing eating behaviors in the Chinese population (Wu et al., 2017). The DEBQ consists of 33 items, including the following three subscales: emotional eating, external eating and restraint eating. Furthermore, the participants also completed the bulimia subscales from the Eating Disorder Inventory-1 (EDI-1) (Lee et al., 1997), 26-item eating attitude test (EAT) (Kang et al., 2017), 21-item Beck depression inventory (BDI) (Shek, 1990) and 20-item self-anxiety scale (SAS) (Zung, 1971) on the day of the scan. The reliability and validity of the above scales have been verified in the Chinese population (Shek, 1990; Lee et al., 1997; Kang et al., 2017; Mo et al., 2020).

### Data Acquisition

A 3.0 T MRI system (Prisma, Siemens, Erlangen, Germany) and a 64-channel phased array coil were used to acquire MRI data. Functional images were obtained using a multislice gradient-echo echo-planar imaging (EPI) sequence as follows: 33 sections with 3.5-mm thickness; 240 time points; repetition time (TR) = 2000 milliseconds; echo time (TE) = 30 milliseconds; field of view (FOV) = 256 mm × 256 mm; matrix = 64 × 64; and flip angle (FA) = 90°. Structural images covering the whole brain were acquired using a 3D magnetization-prepared rapid gradient-echo (3D-MPRAGE) sequence as follows: 192 sections with 1-mm thickness; TR/TE = 2530/2.98 milliseconds; inversion time (TI) = 1100 milliseconds; FOV = 256 × 256 mm^2^; matrix = 256 × 256; and FA = 7°.

We provided patients with earplugs to reduce scanning noise and foam padding to minimize possible head movement. Prior to the examination, subjects were told to close their eyes but not to fall asleep. During the scanning, we reminded the subjects two additional times to prevent them from falling asleep. After the examination, we asked the subjects if they were asleep, and they all said no. The total scanning time is 13 min.

### Data Preprocessing

Functional images were processed using the Resting-State fMRI Data Analysis Toolkit (RESTplus)^1^ and Statistical Parametric Mapping (SPM) 12^2^. The steps for preprocessing data were as follows: (1) the first 10 time points were removed for steady-state magnetization and subject adaptation to scanning noise; (2) slice timing correction; (3) head motion correction; (4) spatial normalization with individual brain images normalized into the Montreal Neurological Institute (MNI) template using the Diffeomorphic Anatomical Registration through Exponentiated Lie algebra (DARTEL) Algorithm (Ashburner, 2007), and the data resampled to 3 mm × 3 mm × 3 mm voxels; (5) smoothing using a 6-mm full-width at half maximum (FWHM) Gaussian kernel to smooth the resulting functional images; and (6) nuisance covariable regression that involved regressing out a total of 26 variables including WM signal, cerebrospinal fluid signal (CSF), and Friston 24-parameter model from the time series of every voxel. Linear detrending was also applied to reduce low-frequency drifts exhibited by blood oxygen level-dependent (BOLD) signals and high-frequency physiological noise (cardiac and respiratory effects). To take possible confounding effects of microhead motion for each subject into consideration, we calculated the mean framewise displacement (FD) that considered the differences in voxelwise movements during its temporal derivation.

In the head motion correction step, data from 2 patients were excluded based on the exclusion criterion (> 2.0 degrees of head rotation or > 2.0 mm displacement). Finally, 29 patients and 29 HCs were included for further analysis.

All the structural data were processed using the Computational Anatomy Toolbox 12 (CAT12)^3^ that was run through SPM12. The structural images were segmented into gray matter (GM), WM, and CSF using the unified segmentation protocol. The GM images were normalized into the MNI space by using the DARTEL algorithm. Then, the GM images were spatially smoothed with a 6-mm FWHM isotropic Gaussian kernel.

### fALFF Calculation

RESTplus has a built-in fast Fourier transform function that converts time series data into the frequency domain and calculates the power spectrum. The ratio of power in the 0.01-0.08 Hz frequency range to the full frequency range (0-0.25 Hz) was calculated (Zou et al., 2008). fALFF values were then z-transformed prior to statistical analyses. All analyses were performed at the whole-brain level.

### Seed-Based Functional Connectivity Analysis

For the FC analyses, ROIs were selected from the clusters in the fALFF maps after two-sample *t* tests. Calculations were made using RESTplus. For each ROI, the seed reference time course was obtained by averaging the time series of all voxels in the ROI. Furthermore, a voxelwise FC analysis was performed by calculating the temporal correlation between the mean time series of the ROIs and the time series of each voxel at the whole-brain level. Fisher z transformation was used to transform individual correlation coefficients into z-values and finally generate an entire brain z-score map for the ROIs of each patient.

### Statistical Analysis

Statistical analysis was performed using SPSS version 26.0 (IBM Corp., Armonk, New York, United States). Demographic and clinical data were compared between BN patients and HCs by two-sample t tests. Two-sample t tests were also conducted to analyze GM differences between the BN and HC groups. Previous studies have indicated that fMRI results can be influenced by changes in brain volume (Oakes et al., 2007). Thus, the results of the brain volume analysis were applied as covariates to subsequent analyses no matter the bivariate outcomes. *P* ≤ 0.05 was considered statistically significant.

Two-sample t tests were performed to identify the group differences in fALFF using the GM mask. Age, educational level, body mass index (BMI), the mean FD and GM volume of each participant were taken as covariates to avoid any undetected effects. We compared fALFF between the two groups by adding BDI and SAS scores to covariates to exclude the effect of depression and anxiety factors. All results were presented at the statistical threshold of *P* < 0.05 by using a false discovery rate (FDR) correction.

Within-group analyses of altered FC patterns were conducted using a one-sample t test, which compared the *z* values of individual voxels with a normal distribution with a mean of zero and an unknown variance. According to Gaussian random field (GRF) theory (Eklund et al., 2016), the results were corrected for multiple comparisons with a 2-tailed voxel *P* < 0.001 and cluster *P* < 0.05.

To determine between-group differences, two-sample t tests were conducted to determine the differences in all possible connections between the BN patients and HCs using the GM mask, controlling for age, educational level, BMI, mean FD values and GM volume. Similarly, we also compared FC between the BN and HC groups by adding BDI and SAS scores as covariates to exclude the effect of depression and anxiety on the results. A GRF correction was performed with 2-tailed voxel *P* < 0.001 and cluster *P* < 0.05 in each functional connectivity analysis.

The partial correlation analysis was performed between FC values of clusters showing between-group differences and DEBQ, EDI-BN and EAT scores in the BN patients controlling for age, educational level, BMI, and mean FD values. Since the BDI and SAS scores of BN patients were higher than those of HCs, we also analyzed the correlation between FC values and scores to detect the possible influence of depression and anxiety factors on FC. *P* < 0.05 was set as the threshold to determine significance.

## Results

### Demographic and Clinical Characteristics of the Participants

Demographic and clinical data are summarized in Table 1. The BN patients and HCs showed no significant between-group differences in demographic data, including age, education, and BMI. The BN patients had higher DEBQ-Emotional, DEBQ-Restraint, EDI-BN, EAT, BDI and SAS scores than the HCs (*P* < 0.001).

**TABLE 1:** Demographic and clinical data of participants.

Variables	Bulimia nervosa (*n* = 29)	Healthy control (*n* = 29)	Statistics	
			t	*p*
Age (y)	22.6 ± 3.4	23.6 ± 1.2	−1.518	0.138
BMI (kg/m^2^)	19.5 ± 2.6	20.0 ± 1.7	−0.796	0.430
Education (y)	15.8 ± 2.4	15.6 ± 2.4	0.222	0.825
DEBQ-Emotional	48.3 ± 11.0	28.2 ± 11.7	6.752	0.000*
DEBQ-Externality	35.1 ± 6.5	32.0 ± 6.2	1.866	0.067
DEBQ-Restraint	37.6 ± 6.8	22.9 ± 5.9	8.748	0.000*
EDI-BN	35.0 ± 5.3	1.8 ± 2.3	30.842	0.000*
EAT	42.9 ± 11.6	12.8 ± 8.5	11.296	0.000*
BDI	18.2 ± 4.2	3.6 ± 2.7	15.740	0.000*
SAS	54.9 ± 12.3	33.8 ± 8.4	7.613	0.000*

In BN group, the age ranged from 16 to 29 years, and the duration of the disorder ranged from 12 months to 20 months. Data presented as the mean ± SD, *P < 0.05. DEBQ, Dutch Eating Behavior Questionnaire; EDI, Eating Disorders Inventory; EAT, eating attitude test; BDI, Beck depression inventory; SAS, self-anxiety scale.

### fALFF Changes in Participants With Bulimia Nervosa

**FIGURE 1:**
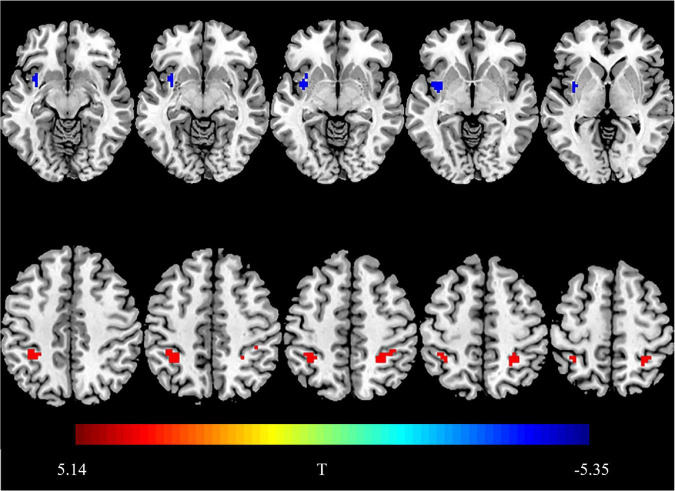
Brain regions showing group differences in fractional amplitude of low-frequency fluctuations. Compared with HCs, the blue area indicates decreased fALFF values in the right insula in BN patients, and the red area indicates increased fALFF values in bilateral IPL. Threshold set at *P* < 0.05, the cluster threshold is 30 (false discovery rate correction).

Compared with the HCs, the BN patients showed decreased fALFF values in the left insula and increased fALFF values in the biliteral inferior parietal lobule (IPL) (*P* < 0.05, FDR corrected) (Figure 1 and Table 2).

**TABLE 2:** Brain regions showing differences in fALFF values in patients with BN compared with HCs.

	Peak MNI coordinates					
Brain regions	X	Y	Z	Cluster size(voxels)	Peak *t* value	*p* value
Left insula	−39	0	−3	36	−5.3549	0.004
Left inferior parietal lobule	−33	−42	45	40	5.1414	0.009
Right inferior parietal lobule	27	−45	54	30	4.5982	0.026

*Brain regions showing differences in fALFF values in patients with BN and HCs. Threshold set at P < 0.05, the cluster threshold is 30 (false discovery rate correction); fALFF, fractional amplitude of low-frequency fluctuation; MNI, Montreal Neurological Institute.*

### Functional Connectivity Patterns in Participants With Bulimia Nervosa

**FIGURE 2:**
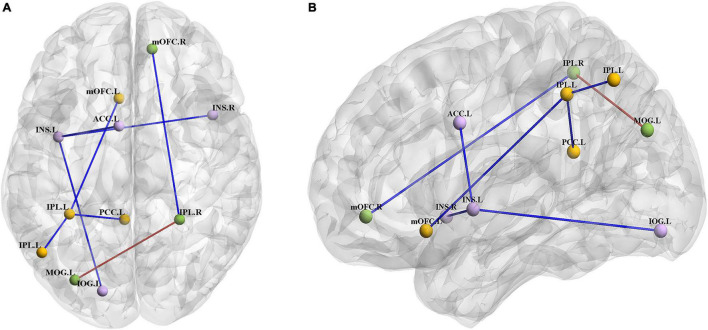
**(A)** Axial view of the brain. **(B)** Left view of the brain. The BN group showed decreased FC between the left INS and the right INS, left IOG and left ACC; between the left IPL and the left IPL, left mOFC and left PCC; and between the right IPL and right mOFC. The BN group showed increased FC between the right IPL and left MOG (*P* < 0.001, GRF corrected). Abbreviations: INS, insula; ACC, anterior cingulate cortex; IOG, inferior occipital gyrus; IPL, inferior parietal lobule; PCC, posterior cingulate cortex; mOFC, medial orbitofrontal cortex; MOG, middle occipital gyrus; L, left; R, right.

**FIGURE 3:**
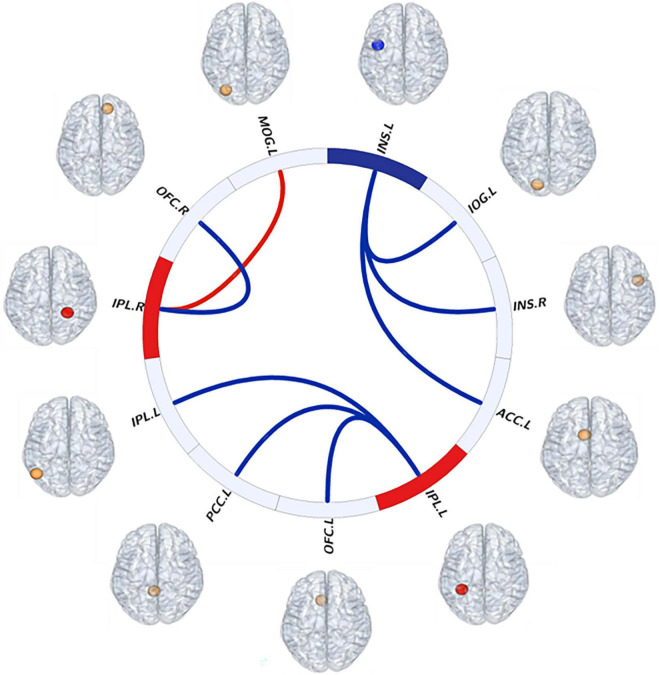
Visual description of resting-state interhemispheric functional connectivity (FC) patterns as evidenced in this “connectome ring.” Red indicates increased fALFF values of brain regions and FC between brain regions. Blue indicates decreased fALFF values of brain regions and FC between brain regions. Abbreviations: INS, insula; ACC, anterior cingulate cortex; IOG, inferior occipital gyrus; IPL, inferior parietal lobule; PCC, posterior cingulate cortex; mOFC, medial orbitofrontal cortex; MOG, middle occipital gyrus; L, left; R, right.

Compared with the HC group, the BN group showed decreased FC between the left insula and the right insula, left inferior occipital gyrus (IOG) and left ACC (Table 3); between the left IPL and the left IPL, left medial orbitofrontal cortex (mOFC) and left PCC (Table 4); and between the right IPL and the right mOFC (Table 5). The BN group showed increased FC between the right IPL and left middle occipital gyrus (MOG) (Figures 2, 3 and Table 5).

**TABLE 3:** Alterations in functional connectivity between the left insula and the other brain regions in patients with BN compared with HCs.

	Peak MNI coordinates				
Brain regions	X	Y	Z	Cluster size (voxels)	Peak t value
Right insula	45	12	−6	46	−4.8818
Left inferior occipital gyrus	−15	−84	−12	44	−4.1051
Left anterior cingulate cortex	−6	6	33	78	−4.5902

*Threshold set at 2-tailed voxel P < 0.001 and cluster P < 0.05 (Gaussian random field correction); The cluster threshold is 38; MNI, Montreal Neurological Institute.*

**TABLE 4:** Alterations in functional connectivity between the left inferior parietal lobule and the other brain regions in patients with BN compared with HCs.

	Peak MNI coordinates				
Brain regions	X	Y	Z	Cluster size (voxels)	Peak *t* value
Left inferior parietal lobule	−48	−63	51	45	−4.0599
Left medial orbitofrontal cortex	−6	21	−12	54	−4.2575
Left posterior cingulate cortex	−3	−45	21	43	−4.3614

*Threshold set at 2-tailed voxel P < 0.001 and cluster P < 0.05 (Gaussian random field correction); The cluster threshold is 40; MNI, Montreal Neurological Institute.*

**TABLE 5:** Alterations in functional connectivity between the right inferior parietal lobule and the other brain regions in patients with BN compared with HCs.

	Peak MNI coordinates				
Brain regions	X	Y	z	Cluster size (voxels)	Peak *t* value
Right medial orbitofrontal cortex	12	48	−6	52	−4.3717
Left middle occipital gyrus	−30	−78	30	42	4.0769

*Threshold set at 2-tailed voxel P < 0.001 and cluster P < 0.05 (Gaussian random field correction); The cluster threshold is 40; MNI, Montreal Neurological Institute.*

### Correlations Between Functional Connectivity Values and Clinical Variables

**FIGURE 4:**
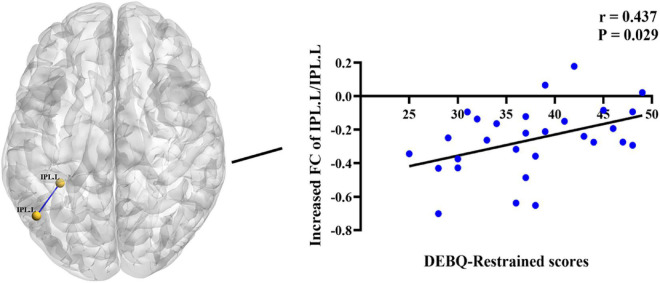
Positive correlations between increased Dutch Eating Behavior Questionnaire (DEBQ)-Restrained scores and increased z values of functional connectivity (FC) between the left IPL and left IPL (*r* = 0.437, *P* = 0.029). IPL.L, left inferior parietal lobule.

In the BN group, the *z* values of internal FC within the left IPL were positively correlated with DEBQ-Restrained scores (*r* = 0.437, *P* = 0.029) (Figure 4). No additional significant correlations were found. We did not observe significant correlations between FC and SAS and BDI scores within the BN group, suggesting that the effects of depressive and anxiety symptoms could be largely excluded.

## Discussion

In the present study that combined fALFF and FC analyses and made comparisons between BN patients and HCs, we found that in the resting state, BN patients had abnormal brain activity and resulting aberrant FC at the whole-brain level, which led to a reorganization of neural networks in BN. The novel findings were as follows: first, there was decreased activation in the left insula, the key node in the BN patients, and FC with the contralateral insula was lower than that in the HCs, indicating that the FC between the bilateral cerebral hemispheres in BN changed. Second, we found that increased activation in the bilateral IPL, which were the key nodes forming a reorganized neural network in BN patients that could modulate other brain regions associated with reward, emotion, cognition, memory and vision functional areas. Third, internal FC within the left IPL was decreased, which was correlated with restrained eating behavior. Our work extended previous findings by providing new useful information for a better understanding of the neural mechanisms underlying BN, with the hope of providing new therapeutic targets involving neuromodulation in BN.

There is much evidence that the posterior parietal cortex, especially the IPL, plays an important role in detecting/maintaining body representations and consciously evaluating these representations (Daprati et al., 2010; Cignetti et al., 2014; Naito et al., 2016; Fontan et al., 2017). In particular, the IPL updates body representations based on the external environment (Naito et al., 2016). Task-based fMRI has suggested that the inferior parietal cortex is consistently involved in the process of response inhibition (Hedden and Gabrieli, 2010; Steele et al., 2013). Furthermore, in populations with high levels of impulsivity, such as hyperactivity disorder, attention deficit disorder, and alcohol use disorder, voxel-based morphometry analyses have shown that the volume of GM in the inferior parietal cortex is decreased (Carmona et al., 2005; Gröpper et al., 2016), suggesting that structural changes in the IPL may lead to impulsive behavior in individuals, and that the structure of a brain region is closely related to corresponding function. Therefore, we have reason to believe that abnormal function activity in the IPL may also lead to impulsive behavior that individuals cannot control. Based on the above research and our results, we believe that the decreased internal FC within the left IPL may underlie cognitive bias, impairments in evaluation of one’s own body representation and impulsive behavior in BN patients. In addition, the increased activity in the bilateral IPL in BN patients may compensate for the decreased internal function of the IPL to some extent, that is, patients need more self-control ability to inhibit their increased impulsive behaviors such as binge eating behavior. In this paper, internal FC within the left IPL correlated with DEBQ-restrained eating behavior scores. One possible explanation is that decreased internal IPL connectivity may influence food intake *via* changes in self-control abilities.

The insular cortex is an anatomical integration hub. As part of the gustatory cortex (Saruco and Pleger, 2021), it is heavily connected with an extensive network of cortical and subcortical brain regions. The insular cortex is thus a site where bodily perception, self-regulatory control, smell and taste processing and afferents from brain regions implicated in emotion processing converge (Gogolla, 2017). The insula is also a key region within the mesocorticolimbic reward pathways, with its external input and expected reward integration function (Gogolla, 2017). Studies have speculated on the relationships between the function of the insular cortex and drug and smoking addictions (Naqvi et al., 2007; Naqvi and Bechara, 2010). Our current results indicated that the BN patients had altered spontaneous brain activity in the left insula and decreased FC with the contralateral insula in the resting state. Combining previous results with those in this study, we speculated that patients with BN have alterations of gustatory processing, disturbances in bodily and self-awareness, and impairments in self-control, leading to inappropriate evaluation of body image and mood-related intermittent binge eating behaviors. At the same time, the altered insula activity in BN patients may also lead to individual reward deficit to some extent. According to reward deficiency syndrome theory (Blum et al., 2000; Luijten et al., 2017), individuals with addictive behaviors have a general deficit in recruiting brain reward pathways, resulting in supposedly reduced pleasurable experience from rewards. To compensate for this reward deficiency, abnormal eating behaviors involving higher levels of food consumption in patients with BN are consequently initiated to satisfy their appetite and the subsequent perception of happiness.

Research to date has demonstrated that patients with BN show abnormal FC at the whole-brain level (Stopyra et al., 2019; Wang et al., 2020). Based on the results of fALFF analysis, we selected the bilateral IPL and right insula as the seeds and found that FC with the mOFC, ACC, PCC, and occipital cortex was different in the BN patients compared with the HCs.

It is well known that the OFC is involved in encoding reward value and decision-making and plays a crucial role in the value attributed to food and subsequent eating behavior (Suzuki et al., 2017; Clarke et al., 2018). In addition, the OFC is a key node in the cognitive and emotional inhibition system (Saruco and Pleger, 2021). Decreased FC between the bilateral IPL and the ipsilateral mOFC may indicate that patients with BN have alterations in the perception of food value and emotional inhibition functions. The PCC receives major inputs from the parietal cortex area and is thereby involved in spatial processing, action in space, and some types of memory (Rolls and Wirth, 2018; Rolls, 2019). McCoy and Platt, 2005 observed that when an individual makes dangerous and uncertain choices, neurons located in the PCC respond accordingly. The PCC is also responsible for processing emotion-related stimuli and participating in certain types of decision-making. Decreased FC between the IPL and PCC may reflect the inability of BN patients to make correct decisions and an interruption of the control process between visceral information integration and memory when faced with food temptation, inducing uncontrollable binge eating behavior. Decreased FC between the IPL, mOFC and PCC may be explained from another perspective. The default mode network (DMN) involves a functionally connected network of brain regions including the IPL, PCC, and mOFC and is thought to be implicated in self-referential processing, which involves monitoring the external environment as well as physical and emotional states (Davey et al., 2016). Decreased FC among the IPL, mOFC and PCC may reflect functional alterations in the DMN in BN patients to some extent, leading to deficits in the sense of self-awareness, distorted body image and lack of recognition of the consequences of binge eating.

The ACC, forming an integral part of the limbic system, has connections with other limbic and related areas involved in emotion and reward-related processing (Rolls, 2019). In addition, the ACC is often considered to belong to the frontal cortex and associated inhibitory control networks (Saruco and Pleger, 2021). Playing a major role in palatable food salience attribution and subsequent decision-making, the ACC was demonstrated to be involved in the regulation of food craving. In line with those results, Atalayer et al. (2014) showed that obese participants exhibited greater FC between the insula and ACC after consuming a full meal. Therefore, in this study, decreased FC between the insula and ACC may indicate impaired salience attribution of palatable food with consequences on food choices in patients with BN, who are still uncontrollably overeating even when they are satiated.

The occipital cortex mediates visual perception of body shape and/or size (Favaro et al., 2012); thus, occipital regions are considered to be relevant to behavioral manifestations of distorted body image (i.e., overestimation of body size, body dissatisfaction and body weight control), which has an important role in the onset and maintenance of eating disorders in patients with BN. Based on these results, we speculate that altered FC between the occipital cortex and other brain regions may lead to the failure of BN patients to correctly judge their own body shape, leading to abnormal eating behaviors and compensatory behaviors such as purging after binge eating.

We acknowledge that the current study has some limitations. First, the sample size was relatively small in this study, and studies with larger sample sizes may help further confirm our conclusions. Second, the menstrual phase has been shown to affect neural activation associated with reward (Dreher et al., 2007). Therefore, future experimental design needs to take into account the possible effects of women’s menstrual cycle on the results. Finally, this study focused only on brain function alterations in BN patients, and additional research is required to extend findings of alterations in brain structure-function coupling in BN at a global level.

## Conclusion

In conclusion, this study demonstrated that even in the resting state, altered regional neural activities and FC were observed in various brain regions. We found that the left insula and bilateral IPL, as the key nodes in the reorganized neural network, had altered FC with other brain regions associated with reward, emotion, cognition, memory, smell/taste, and vision-related functional processing. Furthermore, internal FC with the left IPL was decreased and may be associated with restrained eating behavior. These findings may provide useful information for a better understanding of the neural mechanisms underlying BN and provide more potential therapeutic targets involving neuromodulation, such as the IPL and insula, in patients with BN.

## Data Availability Statement

The original contributions presented in the study are included in the article/supplementary material, further inquiries can be directed to the corresponding authors.

## Ethics Statement

The studies involving human participants were reviewed and approved by Beijing Friendship Hospital Ethical Committee. Written informed consent to participate in this study was provided by the participants’ legal guardian/next of kin.

## Author Contributions

J-NW conceived the framework of the manuscript and implementation and wrote the whole manuscript. L-RT, X-YZ, XS, P-PW, and Z-MY participated in collecting enrolled BN patients and communicating with them. W-HL, QC, and ZCW completed the collection of MRI data of BN patients. G-WW analyzed and processed the magnetic resonance data. PZ participated in the revision and content supplement of the article. Z-JL and ZW revised the layout of the article and checked for grammatical errors. All authors contributed to the article and approved the submitted version.

## Conflict of Interest

The authors declare that the research was conducted in the absence of any commercial or financial relationships that could be construed as a potential conflict of interest.

## Publisher’s Note

All claims expressed in this article are solely those of the authors and do not necessarily represent those of their affiliated organizations, or those of the publisher, the editors and the reviewers. Any product that may be evaluated in this article, or claim that may be made by its manufacturer, is not guaranteed or endorsed by the publisher.

## Footnotes

1
http://www.restfmri.net

2
http://www.fil.ion.ucl.ac.uk/spm

3
http://dbm.neuro.uni-jena.de/cat/
